# Wat1/pop3, a Conserved WD Repeat Containing Protein Acts Synergistically with Checkpoint Kinase Chk1 to Maintain Genome Ploidy in Fission Yeast *S. pombe*

**DOI:** 10.1371/journal.pone.0089587

**Published:** 2014-02-21

**Authors:** Sumit Kumar Verma, Rajeev Ranjan, Vikash Kumar, Mohammad Imran Siddiqi, Shakil Ahmed

**Affiliations:** Molecular and Structural Biology Division, Central Drug Research Institute, CSIR, Sector 10, Jankipuram Extension, Lucknow, India; University of Cambridge, United Kingdom

## Abstract

Aberrant chromosome segregation defects can lead to aneuploidy, a common characteristic of human solid tumors. Aneuploidy is generated due to defects in the mitotic spindle or due to inefficient mitotic checkpoint response. We have isolated a novel mutant allele of *wat1*, a WD repeat containing protein that exhibits conditional synthetic lethality with *chk1* knock out. We observed only a marginal decrease in the level of α tubulin protein level in *wat1-17* mutants after prolong exposure at semi permissive temperature. Interestingly the protein level of α-tubulin was reduced in the *chk1*Δ *wat1-17* double mutant at 18°C with defective microtubule structure. Consistent with loss of microtubule structure in the c*hk1* deletion background, the double mutant of *wat1-17 chk1*Δ was hypersensitive to the microtubule destabilizing agent TBZ suggesting severe defects in microtubule integrity in *wat1-17* mutant in the absence of Chk1. Combination of *wat1-17* with the *chk1* deletion also aggravates the defects in the maintenance of genome ploidy. The mutation in *wat1-17* was mapped to Cys 233 that was changed to tyrosine. Based on the molecular modeling studies, we hypothesize that the substitution of the bulky Tyr residue at Cys233 position in *wat1-17* mutant results in conformational changes. This in turn can affect its intercations with other interacting partners and perturb the overall functions of the Wat1 protein.

## Introduction

Accumulation of mutations and chromosomal aberrations is one of the hallmarks of cancer cells. These aberrations arise due to defects in the genome maintenance mechanisms that include DNA repair and cell cycle checkpoint pathways. Defects in these cell cycle checkpoints result in inappropriate proliferation. DNA damage checkpoints are responsible for maintaining the fidelity of genetic information by arresting cell cycle progression and facilitating DNA repair pathways. Several studies have identified a network of proteins that are involved during the DNA damage checkpoints response. Central to this network are protein kinases of the ATM/ATR family that work as sensors and transducers. These are also known as Tel1/Mec1 in budding yeast and Tel1/Rad3 in fission yeast respectively [1]. Downstream of ATM and ATR are effector molecules Chk1 and Chk2 respectively. These are serine threonine kinases that sense DNA damage and phosphorylate a number of proteins that regulate cell cycle progression and DNA repair pathways [2]. ATR is the major upstream kinase that phosphorylates and activates Chk1 [3]–[7]. Chk1, an evolutionarily conserved protein kinase is an essential component of the DNA damage checkpoint [8]–[10]. In response to DNA damage, the protein kinase Chk1 is phosphorylated and inhibits mitotic entry by phosphorylating Wee1 and Cdc25 to prevent activation of Cdc2 [11].

The spindle assembly checkpoint blocks chromosome segregation until all the chromosomes are attached to the mitotic spindle. The anaphase-promoting complex (APC), a multi-subunit E3 ubiquitin ligase is required for the degradation of both cyclin B and cohesin to promote metaphase to anaphase transition. The activation of Mad2, a spindle assembly checkpoint protein prevents the association of APC with Slp1/Cdc20 and blocks the cells during metaphase until all the chromosomes are properly attached to the mitotic spindle [12]. Involvement of Chk1 pathway to delay metaphase to anaphase transition in response to DNA damage has also been shown in *S. pombe* and *Drosophila* [13], [14].

The WD40-repeat motif was identified originally in the β-subunit of heterotrimeric G proteins [15] and subsequently has been found in a wide spectrum of regulatory proteins, where it functions in mediating protein-protein interactions. WD40-repeat proteins adopt a β-propeller structure, which can use one or two blades to interact with other proteins without affecting the other blades [16], [17]. It is assumed that one (or more) WD repeat within a given protein specifically interacts with different partner proteins, thus creating multiple protein–protein interactions [18].

Fission yeast Wat1/pop3 is a homologue of Lst8 of budding yeast. Depletion of Lst8 in budding yeast cells results in a rapid arrest of cell growth [19], [20]. The budding yeast LST8 functions in the delivery of Gap1 protein, and possibly other amino acid permeases, from the Golgi to the cell surface [20]. A mutant allele of LST8 (*lst8-1*) exhibited synthetic lethality with the *sec13-1* mutation [20]. Fission yeast Wat1 has been shown to play an important role in the establishment of actin and microtubule cytoskeleton [21]. The role of Wat1 in mRNA maturation and its requirement for the maintenance of genome stability and microtubule integrity has been well studied [22]. Upon nutrient starvation, the *wat1* mutant cells fail to arrest in the G1 phase and hence are sterile in fission yeast [21], [23]. Mammalian LST8 is a functional component of mTOR signaling complex and interacts with the kinase domain of mTOR to stabilize its interaction with raptor. It also participates in regulating cell growth through the mTOR S6K1 signaling pathway. Recently the truncated mTOR (kinase domain) and LST8 protein have been co-crystallized. This has helped for the understanding of mTOR function and its inhibition by rapamycin and ATP competitive compounds [24].

In a genetic screen to identify conditional synthetic lethal mutants with *chk1* null mutant, a novel temperature sensitive mutant allele of *wat1* was isolated. Our study shows that the *chk1* null mutant and *wat1* mutant together pose a grave effect on the survival of fission yeast cells, when exposed to semi-permissive temperature and the microtubule destabilizing agent, thiabendazole. The protein level of α tubulin was slightly reduced in *chk1* delete *wat1-17* double mutant at permissive temperature which further decreased after shifting these cells to semi-permissive temperature. The double mutant of *wat1-17 chk1* delete cells exhibit high incidence of polyploidy at semi-permissive temperature. Homology modeling and structural prediction studies involving the Wat1 protein identified seven WD repeats consisting of β-sheets. The C233Y mutation in the *wat1-17* mutant was located in the sixth repeat and has been shown to affect its interaction with the Prp2 protein.

## Materials and Methods

### Strains and Growth Conditions

*Schizosaccharomyces pombe* strains used in this study are listed in Table 1. Standard genetic methods were utilized for making strains as described [25]. For temperature-shift experiments, cells were grown to mid log phase at 25°C and then shifted to restrictive temperature. For spotting experiments, cells were grown at 25°C up to mid log phase, 10^7^ cells were serially diluted and spotted on required plate. For temperature sensitivity experiments plates were incubated at indicated temperature. For TBZ sensitivity assay, mid log phase grown culture were serially diluted, spotted on plates containing 10 ug/ml thiabendazole and incubated at 25°C. For diploidisation studies strains were plated on rich medium containing phloxine B and incubated at 25°C for 3–4 days.

**Table 1:** Strains used in this study.

Strain	Genotype	Source
SP6	*h^−^ leu1-32*	Lab stock
NW158	*h^+^ leu1-32 ura4D18 chk1::ura4 ade6-216*	Nancy Walworth
SH46	*h^+^leu1-32 ts17/wat1-17*	This study
SH116	*h^+^ leu1-32 ura4D18 wat1-17 chk1::ura4 ade6-210*	This study

### Microscopy and Indirect Immunofluorescence Studies

For staining nucleus, cells were grown to mid log phase at 25°C. Then shifted to 18°C, samples were collected, fixed with 70% ethanol and stained with DAPI, visualized using a fluorescence microscope. About 200 cells were counted for abnormal DAPI staining bodies and percentage was calculated. Immunofluorescence studies were performed using exponentially growing cells essentially as described earlier [26]. Microtubules were detected using anti α- tubulin antibody at a 1∶50 dilution, incubated overnight at room temperature with rotation, washed, and then detected with secondary antibody coupled to Alexa fluor488 (Life technologies) at a dilution of 1∶100 and incubated at room temperature for 4 h. The cells were analyzed using a fluorescence microscope and processed by using Adobe Photoshop.

### Identification of Gene by Complementation Analysis

In order to identify the gene coding for *ts17* mutant, the *S. pombe* genomic DNA library was transformed in *ts17/wat1-17* mutant strain by lithium acetate method [25]. Leu^+^ Ts^+^ transformants were obtained and plasmid was recovered from Leu^+^ Ts^+^ co-segregants. Restriction digestion and sequencing analysis identify 11 kb region on chromosome II containing four open reading frames coding for *inp2*, *wat1/pop3*, *nrf1* and SPBC21B10.03c genes. Further 3.3 kb *Hind*III fragment containing full length *wat1/pop3* gene was re-transformed in *ts17/wat1-17* mutant strain for complementation analysis.

### Cloning of Mutant *wat1* Gene by Gap Repair Method

Mutant *wat1* gene (*wat1-17*) was cloned by the gap repair method. Wild type *wat1* gene cloned in pSP1 vector, was digested with *Nde*I and *Nco*I restriction enzyme that deleted 2524 bases from upstream to downstream of *wat1* coding region. The linear fragment containing upstream and downstream sequences of *wat1* gene with a leucine selectable marker was isolated by gel elution and transformed in *wat1-17* mutant strain. Transformants containing plasmid with mutant allele of *wat1* gene (*wat1-17*) was distinguished from those with integrated plasmid by the mitotic stability of leucine marker present on the plasmid. Plasmid containing mutant copy of *wat1* gene (*wat1-17*) was recovered from the transformant as described [25] and was confirmed by *Hind*III digestion. The complete gene coding for *wat1* mutant (*wat1-17*) was sequenced using appropriate primers and was compared with the wild-type sequence of *wat1^+^* gene.

### Preparation of Whole Cell Lysate and Western Blot Analysis

Cells were grown up to mid log phase at 25°C then shifted at 18°C. Cells were harvested by centrifugation at indicated time interval and lysed using glass beads and a Fast Prep (Bio 101) vortex machine. Lysate in Phosphate Buffered Saline (PBS) was centrifuged at 10000 rpm in a microfuge for 5 min at 4°C. Supernatant was collected and protein estimation was performed using the Bradford assay method. For western blot analysis, 100 µg of total cell lysate was run on 10% SDS-PAGE, transferred to nitrocellulose membrane and probed with anti α-tubulin (Sigma, cat no. T6199) and anti-Cdc2 antibodies. A peroxidase-coupled secondary antibody and the enhanced chemiluminescence detection system (Millipore) were used to detect the immune complexes.

### Flow Cytometry

Aliquots of 10^6^ cells were collected from mid log phase cultures, fixed in 1 ml of 70% ethanol before storing at 4°C. For flow cytometry, the cells were rehydrated by washing with 3 ml of 50 mM sodium citrate, resuspended in 0.5 ml of 50 mM sodium citrate containing 0.1 mg/ml RNase A, and incubated at 37°C for 2 h. Cells were stained by adding 0.5 ml of sodium citrate solution containing 10 µg/ml Propidium Iodide and stored at 4°C in dark for 1 hour and subjected to flow cytometry as described earlier [27]. Just prior to flow cytometry, samples were sonicated to avoid inaccurate readings resulting from the clumping of cells. Samples were analyzed with a Becton-Dickinson FACS Calibur.

### Yeast Two-hybrid Analysis

For two-hybrid interaction studies *prp2*, *wat1^+^* and *wat1-17* mutant genes were amplified and cloned in pACT2 and pAS2 vector respectively using forward primer 5′-GATCCCATGGATTT GTCTTCCAGATTATC-3′, reverse primer 5′-GATCGGATCCATCACCATGCATTAGCTTT ATAG-3′ for *prp2* and forward primer 5′-GATCCATATGTCAGTACAGTATCCACCA-3′, reverse primer 5′-GATCGGATCCACTTAAATTTGGTAGTCATTAAG-3′ for *wat1* gene. Plasmid containing Prp2 as prey fused with the GAL4 activation domain (pACT2) and the Wat1 protein fused to the DNA-binding domain of the GAL4 transcription factor were co-transformed in PJ69-4A strain (Clontech). Interaction studies were performed using LacZ and HIS3 as reporter gene on SD-leu-trp plates containing X-gal, or lacking histidine respectively.

### Molecular Modeling

Homology modeling procedure was followed for construction of Wat1 model. Initially suitable templates were searched using BlastP tool against PDB database. Recently solved crystal structure (PDB-ID, 4JSP) of human mTORDeltaN-mLST8-ATPgammaS-Mg complex [24] was taken as template to build models of Wat1. From this complex, LST8 co-ordinate information was utilized. Clustalw2 omega (http://www.ebi.ac.uk/Tools/msa/clustalo/) was used to generate the query template alignment, which served as input for homology modeling program Modeller9v10 [28]. We generated 20 models, which were submitted to SAVS server for structure verification. A model of mutant Wat1 was also constructed with the help of UCSF Chimera [29]. For molecular visualization Chimera was used. Interactive alignment was generated with the help of ESPript [30].

## Results

### Isolation and Identification of Synthetic Lethal Mutants with *chk1* Null Mutants

**Figure 1:**
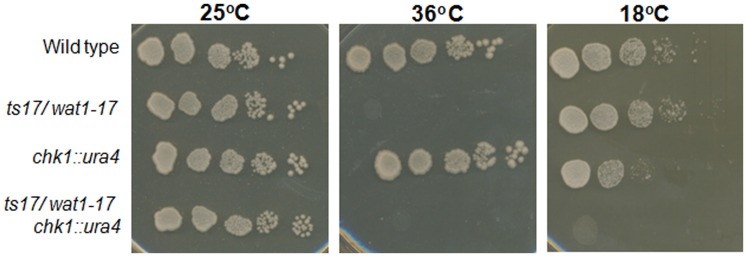
The *ts17/wat1-17* mutant allele exhibit conditional lethality with *chk1* knockout. Indicated strains were grown at 25°C, serially diluted and spotted on YEA plates. Plates were incubated at indicated temperature for 3 days except 18°C plate that was incubated for 7 days before taking photographs.

During a temperature sensitive screenings with *chk1* null mutant background, five conditional mutants were isolated that were unable to form colonies at semi permissive temperature in *chkl* knockout background as described earlier [31]. The *ts17* mutant strain, isolated from the same genetic screen was back-crossed with wild type strain and a pure *ts17^−^* mutant strain was isolated. The *ts17^−^* mutant cells exhibited temperature sensitive phenotype and were unable to form colonies at 36°C. To further confirm the conditional lethality associated with the *chk1* knockout, we checked the ability of *ts17/wat1-17* and *chk1Δ ts17/wat1-17* double-mutant strain to form colonies at 18°C. The *ts17/wat1-17* single mutant were able to grow at 18°C but in *chk1* deletion background the *ts17/wat1-17* mutant cells were unable to form colonies (Figure 1) indicating a conditional synthetic lethality of *ts17/wat1-17* with *chk1* knock out.

The gene coding for the *ts17/wat1-17 m*utation, was identified by complementation analysis as described in experimental procedures. Sequencing and database analysis identified the 11 kb region on chromosome II containing four open reading frames coding for *inp2*, *wat1/pop3*, *nrf1* and SPBC21B10.03c gene respectively. Further subcloning identified a 3.3 kb *Hind*III fragment containing full length *wat1/pop3* gene was sufficient for the complementation of the temperature sensitive phenotype of *ts17/wat1-17* mutation (data not shown).

### Combination of *wat1-17* Mutant with *chk1* Knockout Renders the Cell Sensitive to Microtubule Destabilizing Agent

**Figure 2:**
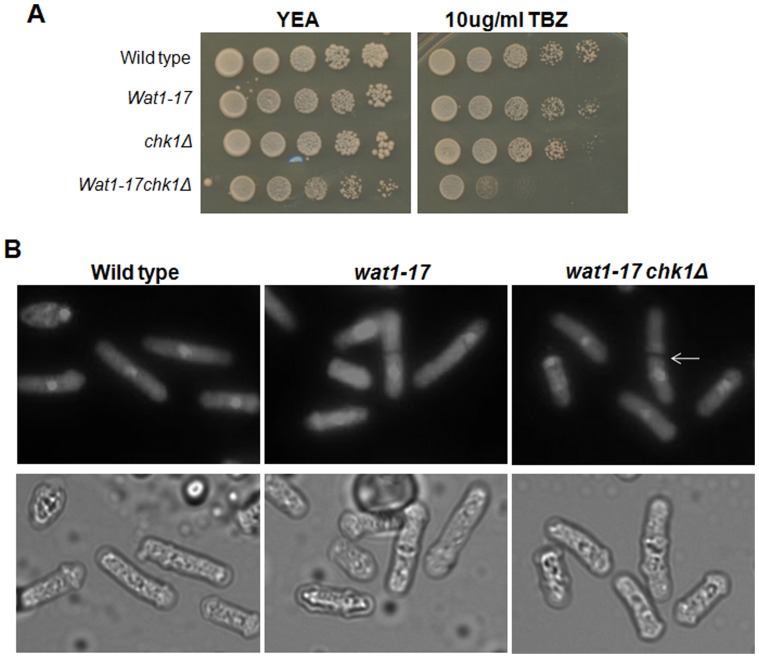
The *wat1-17 chk1* delete cells are hypersensitive to microtubule destabilizing agent. **A.** Indicated strains were grown at 25°C, serially diluted and spotted on YEA plate or plate containing 10 ug/ml thiabendazole. Plates were incubated at 25°C for 3-4 days before taking photographs. **B.** Indicated strains were grown till mid log phase at 25°C and then shifted at 18°C for 36 hr, fixed with 70% ethanol and stained with DAPI. About 250 cells were counted for the presence of aberrant nuclei and percentage was calculated. Scale bar: 10 µm.

Earlier studies have shown α-tubulin reduction and actin disorganization in *wat1* mutants [21], [22]. Wat1 protein has also been shown to be required for the maintenance of microtubule integrity. To further explore the role of *wat1-17* mutant allele in microtubule stability, we tested the sensitivity of *wat1-17* mutant with microtubule destabilizing drug. Contrary to earlier studies [22] we observed that the mutant allele of *wat1-17* was not sensitive to microtubule destabilizing drug (Fig. 1B). Interestingly *chk1*Δ *wat1-17* double mutant was hyper-sensitive to tubulin destabilizing agent and was unable to form colonies on plate containing thiabendazole (Fig. 2A) indicating a possible requirement of Chk1 for the recovery of *wat1-17* mutant cells under defective microtubule condition. The previously [22] isolated *wat1-5235* mutant is cold sensitive while the novel *wat1-17* mutant is not, suggesting that the *wat1-5235* mutation affects the function of Wat1 protein more severely than the *wat1-17* mutation. We also monitored the cellular morphology of *wat1-17 chk1Δ* double mutant along with the *wat1-17* single mutant at semi permissive temperature by staining the nuclei with DAPI. After 48 hr incubation at 18°C abnormal mitosis as defined by more than one DAPI -stained body was observed in about 8% of the *wat1-17 chk1Δ* cells while only <1% cells of the *wat1-17* single mutant exhibited such abnormal nuclei (Fig. 2B) indicating severe defect in *wat1-17 chk1Δ* mutant.

### Tubulin Level was Reduced in *chk1Δ wat1-17* Double Mutant as Compare to *wat1-17* Single Mutant

**Figure 3:**
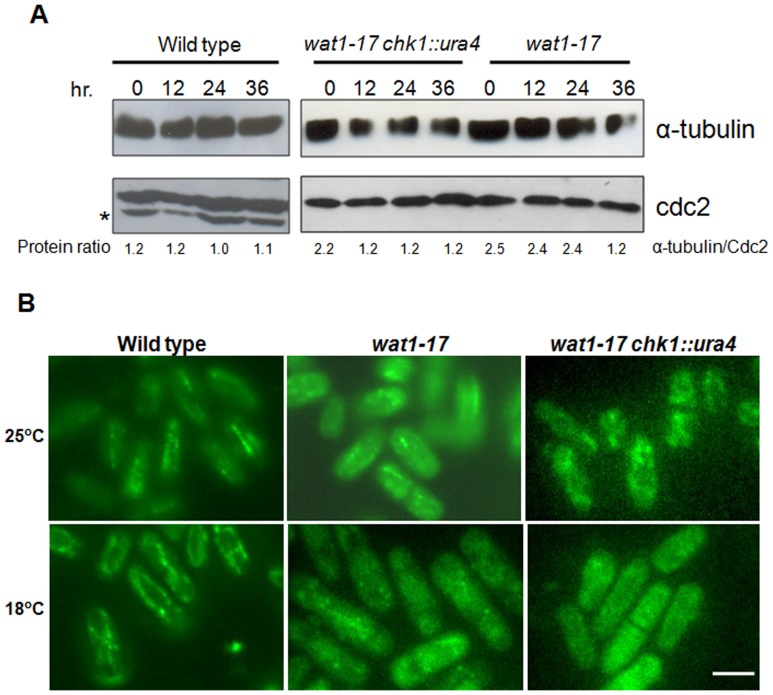
The *wat1-17 chk1* delete cells shows reduced α tubulin levels and defects in mictrotubule structure. **A.** The wild type, *wat1-17* and *wat1-17 chk1*Δ cells were grown at permissive temperature till mid log phase then shifted at 18°C for indicated time. Protein lysate was prepared as described in material and methods, samples were run on 10% SDS PAGE, transferred on nitrocellulose membrane and probed with anti α-tubulin antibody. Anti-cdc2 antibody was used as loading control. Signals were quantitated on Gel Doc system (Life Technologies) and protein ratio was calculated. The asterisk indicates a non specific band. **B.** Indicated strains were grown at 25°C and shifted at 18°C for 48 hr. Cells were processed for immunoflourescence microscopy using anti α- tubulin antibody. Scale bar: 10 µm.

Previous work has identified Wat1 as a protein that is required for the maintenance of α-tubulin level [22]. To explore the effect of *wat1-17* mutant allele on expression of α-tubulin, we examined the level of α-tubulin after shifting the *wat1-17* mutant cells to the non-permissive temperature. We did not observed reduction in α-tubulin protein level at 36°C (data not shown) but there was reduction in the level of α-tubulin protein after shifting the *wat1-17* mutant cells to 18°C for 36 hr (Fig. 3A). Interestingly there was about 50% reduction in the protein level of α-tubulin in *chk1*Δ *wat1-17* double mutant as compare to *wat1-17* single mutant just after 12 hr shift at 18°C (Fig. 3A). Consistent with the reduced α-tubulin level in *chk1* deletion background, the double mutant of *wat1-17 chk1*Δ were hypersensitive to microtubule destabilizing agent (Fig. 2A) suggesting severe defects in microtubule integrity in *wat1-17* mutant in the absence of Chk1.

### Microtubule Structure are Compromised in the *wat1-17* Mutant at Semi Permissive Temperature

Reduction of α -tubulin protein levels, in *wat1-17* and *wat1-17 chk1* delete cells prompt us to monitor the microtubule structure at permissive and semi permissive temperature. Immunofluorescence microscopy with anti α-tubulin antibody showed normal microtubule structure in wild type cells at 25°C and 18°C (Fig. 3B left, upper and lower panel). Interestingly *wat1-17* single mutant cells grown at 25°C have shorter microtubules (Fig. 3B, upper, middle panel) while at semi permissive temperature very few shorter microtubules were present (Fig. 3B, lower middle panel) indicating compromised microtubules at 18°C. In *wat1-17 chk1*Δ double mutant very few short microtubules were observed at permissive temperature (Fig. 3B, upper right panel) that were absent once the cells were shifted at semi permissive temperature (Fig. 3B, upper lower panel) indicating a severe defect in microtubule structure at 18°C. Hypersensitivity of *wat1-17 chk1*Δ double mutant with TBZ (Fig. 2A) is in agreement with the severe defects in microtubule structure in the double mutant.

### Combination of *wat1-17* with the *chk1* Deletion Aggravates the Defects in Maintenance of Genome Ploidy

**Figure 4:**
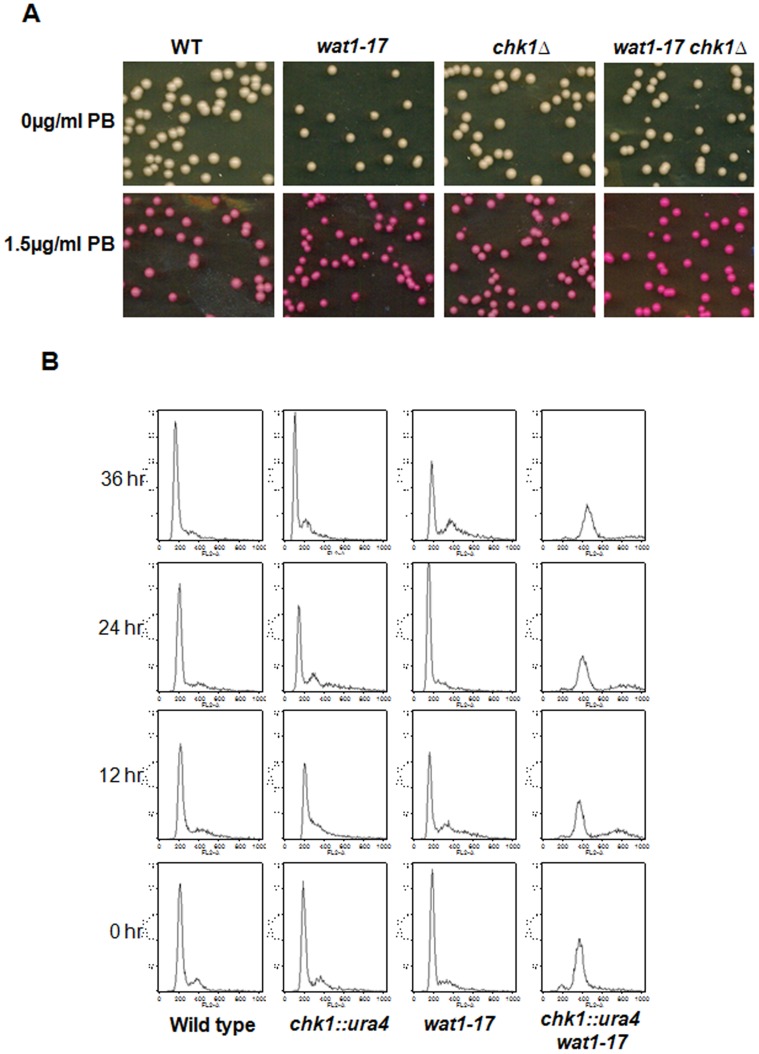
The diploidisation of *wat1-17* and *wat1-17 chk1Δ* strain. **A.** Wild type, *wat1-17*, *chk1Δ* and *wat1-17chk1*Δ double mutant were grown up to mid log phase, about 1000 cells were spread on YEA plates containing 1.5 µg/ml Phloxine B. All the plates were incubated at 25°C for 3–4 days before taking photographs. **B.** FACS analysis of wild type, *chk1*Δ, *wat1-17, wat1-17chk1*Δ mutants. The asynchronous cultures were grown at 25°C and shifted to 18°C, samples were taken at 12 h interval, fixed and stained with the propidium iodide. Samples were analyzed for BD FACS caliber for DNA content analysis.

In earlier studies the *wat1* mutant has been shown to required for the maintenance of genome ploidy and chromosome stability [22]. We used Phloxine B to observe the diploidising property of *wat1-17* mutant cells. Phloxine B, a xanthene dye with a red colour often used to distinguish diploid strains of fission yeast from haploid strains and has been used to detect the genome duplication [22], [32]. The result shows that the colonies from *wat1-17* and *wat1-17 chk1*Δ strains were slightly dark colored on plates containing Phloxine B as compared to wild type and *chk1*Δ cells, indicating the presence of diploid cells in these strains (Fig. 4A).

To observe the polyploidy in detail, DNA content of *wat1-17, chk1*Δ, *wat1-17chk1*Δ mutants was measured by flow cytometry. The strains were grown at 25°C till mid log phase then shifted at semi permissive temperature (18°C), samples were collected and processed for FACS analysis. At 25°C the wild type, *wat1-17* and *chk1*Δ cells exhibited the normal ploidy of diploid cells at each time point while most of the cells of the *wat1-17 chk1*Δ double mutant exhibited an increase in ploidy from 2N to 4N (Fig. 4B) indicating that the double mutant could be partially defective in mitosis even at the permissive temperature. More importantly the DNA peak in *wat1-17 chk1*Δ shifted towards polyploidy when these cells were shifted to 18°C (Fig. 4B) indicating severe defects in maintenance of genome ploidy in the double mutant.

### Mapping and Identification of *wat1-17* Mutation by Gap Repair

**Figure 5:**
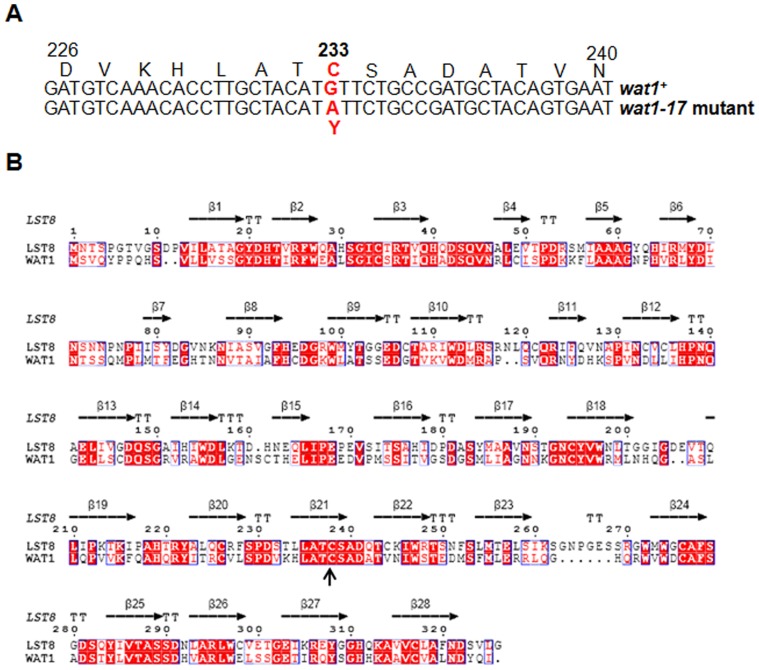
Mapping of *wat1-17* mutation and its conservation with human Lst8. **A.** Location of mutation in *wat1-17* gene. **B.** ESPript generated sequence alignment of Wat1 and human Lst8. Secondary structure assignment was according to crystal structure Lst8 (PDB-ID, 4JSP).

To identify the mutation in *wat1* gene we cloned the *wat1-17* mutant gene by gap repair as described in material and methods. Sequencing and comparison with wild type sequence of *wat1^+^* gene indicated a mutation from nucleotide G to A, that changes amino acid Cysteine to Tyrosine at position 233 in Wat1 protein (Fig. 5A). Multiple sequence alignment studies show that Cysteine residue at 233 is conserved in yeast and human (Fig. 5B) indicating that this residue might be having important role in Wat1 function.

### Mapping of *wat1-17* Mutation based on Homology Modeling

**Figure 6:**
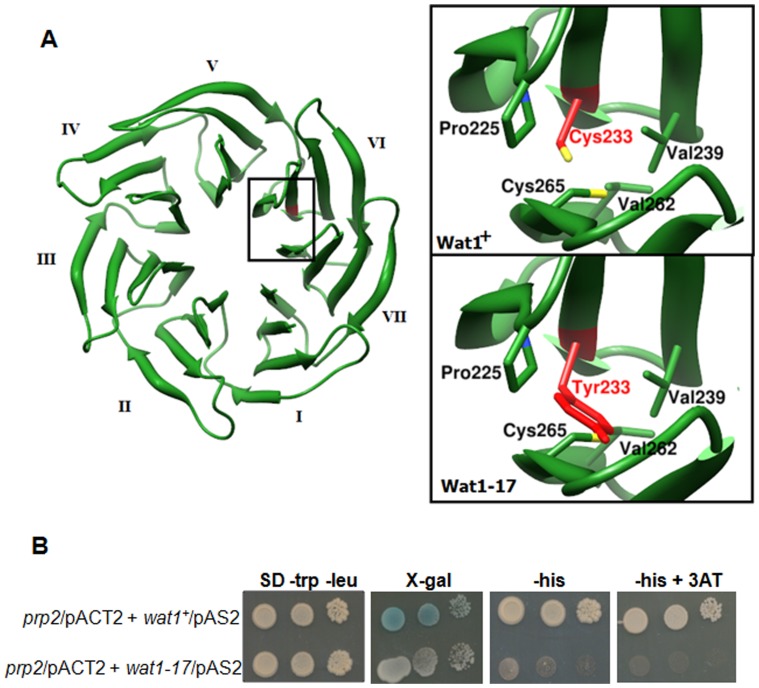
Molecular Modeling analysis of Wat1 and its interaction with Prp2. **A.** 3D model of *S. pombe* Wat1 showing heptad WD repeats. Close view of region of interest where C233Y mutation lies. Upper panel shows wild type Wat1 having Cys 233 (colored in red). Lower panel shows model of mutant Wat1 having Tyr at 233 position(colored in red). Images were generated with the help of Chimera1.6. **B.** The Wat1 mutant protein fails to interact with Prp2 in a yeast two hybrid interaction assay. Prp2 Protein was used as prey, fused with activation domain (pACT2) and the Wat1 or Wat1 mutant protein was fused to the DNA-binding domain (pAS2) as bait. Interaction was analyzed using LacZ as reporter gene on SD-trp-leu plates containing X-gal and HIS marker as a reporter gene on SD-trp-leu plate lacking histidine. 3AT was used to prevent any leaky expression of HIS marker gene.

To identify the structrural basis for the function of the Wat1-17 mutant, homology modeling was perfomrd as described in material and method. Sequence alignment revealed that Wat1 has significant sequence identity (∼47%) with human Lst8 (Fig. 5B). Wat1 model depicted seven WD repeats consisting of only β-sheets (Fig. 6A, left). Overall structure appeared as β-propeller, where each repeat has four β-strands arranged in antiparellel fashion. Structural superimpostion of Wat1 with Lst8 resulted in less than 0.5 Å root mean square deviation (rmsd), which confirms its relatedness at the structural level. In Wat1 model, we were more interested in exploring the position of C233Y mutation, which was found to be located in sixth repeat (Fig. 6A right). We hypothesize that the bulky nature of Tyrosine side chain at position 233 in *wat1-17* mutant could alter the conformation of Wat1 protein (Fig. 6A, right, compare upper panel with lower panel) and hence affect the overall function of the protein.

### The Mutant Wat1 Protein was Unable to Interact with Prp2

We further test the hypothesis that the substitution of Tyrosine residue at position 233 of Wat1-17 protein could affect its interaction pattern with their known interacting partners. Prp2 is the large subunit of U2AF and is required for pre-mRNA splicing [33], [34]. Wat1 was isolated as interacting partner of Prp2 in a two hybrid screen [22]. Mutation in the *prp2* (also known *mis11*) gene leads to the loss of mini-chromosomes indicating an important role of Prp2 in maintaining genomic stability [35]. We tested the interaction of Wat1-17 mutant protein with Prp2 by yeast two hybrid assays. As reported earlier [22] the strains expressing wild type copy of Wat1 and Prp2 protein produced blue color on plates containing X-gal and were able to form colonies on plate lacking histidine (Fig. 6B) suggesting a positive interaction between two proteins. More interestingly cells expressing *wat1-17* mutant protein and Prp2 protein were unable to produce blue color on plates containing X-gal and were unable to form colonies on plates lacking histidine (Fig. 6B) indicating the loss of interaction due to mutation in Wat1 protein.

## Discussion

A complex haploinsufficient screening with the *chk1* knockout was carried out to identify the genes related to checkpoint function. This led to the identification of a *ts17* mutant that code for the *wat1* gene. Wat1 is a highly conserved protein that consists of seven WD repeats [18]. Budding yeast *lst8*, a homolog of *wat1* is an essential gene for survival and acts as a positive regulator of the TOR complex [20], [36]. Wat1 is also known to interact with Prp2, the large subunit of the essential splicing factor U2 auxiliary factor [23]. The conditional synthetic lethality of *wat1-17* with *chk1*Δ indicates the requirement of Chk1 for the recovery of *wat1-17* mutant cells at semi permissive temperature (18°C). The reduction in the number of shorter microtubules in the *wat1-17* mutant at semipermissive temperature could be due to the loss of cytoplasmic microtubules at low temperature as previously reported [37], [38]. In the absence of Chk1, loss of microtubules might affect the survival of the cells due to the loss of spindle checkpoint as Chk1 has been linked with spindle checkpoint pathway in yeast and human cells [13], [39]. There is another possibility that the reduction of the α-tubulin protein level in *wat1-17 chk1*Δ could result in shorter microtubules at 18°C. This could lead to chromosome segregation defects. In fact, the sensitivity of the *chk1* deletion *wat1-17* double mutant towards the microtubule destabilizing drug, TBZ suggests a possible requirement of Chk1 for the recovery of *wat1-17* mutant cells under defective microtubule conditions. However only 8% chromosome segregation defect in double mutant does not coincide with the loss of survival at semi-permissive temperature, suggesting that the reduced viability at 18°C in *wat1-17 chk1Δ* cells could be due to the defects in additional pathway such as stress response as Wat1 protein has been shown to interact with the components of TOR complex [40]. Target of Rapamycin (TOR), an evolutionally conserved phosphatidylinositol kinase –related protein controls cell growth in response to nutrients and growth factor.

At 18°C *wat1-17* mutant exhibits genome diploidising defects as it fail in cell division after genome duplication. The broader DNA peak in *wat1-17chk1*Δ cells at the semi permissive temperature indicates increase in ploidy. Increase in ploidy could be due to the chromosome segregation defect that has been visualized in the form of increased aberrant nuclei in the *wat1-17chk1*Δ double mutant as compared to the single mutant. Two classes of genes have been implicated for the maintenance of ploidy. The first class of mutants is defective in regulating DNA replication and allows re-replication within one cell cycle [41], [42]. The other class of mutants exhibit increase in ploidy and chromosome segregation defects due to the defects in spindle pole body duplication, kinetochore attachment and microtubule formation [43]–[46]. The *wat1-17 chk1*Δ double mutant falls in the second class of mutants that posses significant defects as evidenced by the reduction in α-tubulin protein level, shorter microtubule structure, as well as a majority of the cells exhibiting increase in ploidy.

The protein kinase Chk1 is a well-established signal transducer in the DNA damage checkpoint. Recent studies have presented evidence to indicate that Chk1 also plays a critical role in the spindle checkpoint [13], [39] and has also been implicated to delay metaphase to anaphase transition in *S. pombe* and Drosophila [31], [13], [14]. Chk1 has been shown to be required for the mitotic arrest in response to taxol treatment, a drug that stabilizes microtubules [47]. Genetic interaction studies have identified that Msc1, a multi-copy suppressor of Chk1, promotes cell survival in the absence of Chk1 and also that it requires an intact mitotic spindle checkpoint [48], [49]. In the same series, the work presented here further emphasizes the requirement of Chk1 in response to defective microtubule and suggests a possible role for Chk1 in the mitotic spindle checkpoint pathway. However further work need to be done to strengthen our understanding of the spindle checkpoint involving Chk1 and Wat1.

The mutation in the *wat1-17* mutant allele was found to be located at position 233 in the sixth repeat. This mutation changes the Cysteine residue to Tyrosine. Structural analysis suggests that the bulky nature of Tyrosine side chain in the *wat1-17* mutant could alter the overall conformation of Wat1. This can then affect its interaction with other proteins and hence affect its function. Less likely alternate possibility is that the adjacent Cysteine residue at 265 position could be responsible for the formation of disulfide bond with Cys233. The presence of Tyrosine at this position in the *wat1-17* mutant can result in the disruption of this disulfide bond, this in turn can affect the overall function of the Wat1 protein. In agreement with our hyphothesis the Wat1-17 mutant protein was unable to interact with Prp2 suggesting that the bulky nature of Tyrosine residue indeed affects its interaction with the partner.

## References

[1] McGowan C, Russell P (2004) The DNA damage response: sensing and signaling. Curr Opin Cell Biol 16: 629–633. 15530773 10.1016/j.ceb.2004.09.005

[2] Nyberg K, Michelson R, Putnam C, Weinert T (2002) Toward maintaining the genome: DNA Damage and Replication Checkpoints. Annu Rev Genet 36: 617–656. 12429704 10.1146/annurev.genet.36.060402.113540

[3] Feijoo C, Hall-Jackson C, Wu R, Jenkins D, Leitch J, Gilbert D, et al (2001) Activation of mammalian Chk1 during DNA replication arrest: a role for Chk1 in the intra-S phase checkpoint monitoring replication origin firing. J Cell Biol 154: 913–923. 11535615 10.1083/jcb.200104099 PMC1255922

[4] Heffernan T, Simpson D, Frank A, Heinloth A, Paules R, et al (2002) An ATR- and Chk1-dependent S checkpoint inhibits replicon initiation following UVC-induced DNA damage. Mol Cell Biol 22: 8552–8561. 12446774 10.1128/MCB.22.24.8552-8561.2002 PMC139882

[5] Liu Q, Guntuku S, Cui X, Matsuoka S, Cortez D, et al (2000) Chk1 is an essential kinase that is regulated by Atr and required for the G(2)/M DNA damage checkpoint. Genes Dev 14: 1448–1459. 10859164 PMC316686

[6] Shiloh Y (2003) ATM and related protein kinases: safeguarding genome integrity. Nat Rev Cancer 3: 155–168. 12612651 10.1038/nrc1011

[7] Zhao H, Piwnica-Worms H (2001) ATR-mediated checkpoint pathways regulate phosphorylation and activation of human Chk1. Mol Cell Biol 21: 4129–4139. 11390642 10.1128/MCB.21.13.4129-4139.2001 PMC87074

[8] Walworth N, Davey S, Beach D (1993) Fission yeast chk1 protein kinase links the rad checkpoint pathway to cdc2. Nature 363: 368–371. 8497322 10.1038/363368a0

[9] Al-Khodairy, Fotou F, Sheldrick K, Griffiths D, Lehmann A, et al (1994) Identification and characterization of new elements involved in checkpoint and feedback controls in fission yeast. Mol. Biol. Cell 5: 147–160. 10.1091/mbc.5.2.147 PMC301021 8019001

[10] Walworth N, Bernards R (1996) rad-dependent responses of the chk1-encoded protein kinase at the DNA damage checkpoint. Science 271: 353–356. 8553071 10.1126/science.271.5247.353

[11] Rhind N, Furnari B, Russell P (1997) Cdc2 tyrosine phosphorylation is required for the DNA damage checkpoint in fission yeast. Genes Dev 11: 504–511. 9042863 10.1101/gad.11.4.504

[12] Bharadwaj R, Yu H (2004) The spindle checkpoint, aneuploidy, and cancer. Oncogene 23: 2016–2027. 15021889 10.1038/sj.onc.1207374

[13] Collura A, Blaisonneau J, Baldacci G, Francesconi S (2005) The fission yeast Crb2/Chk1 pathway coordinates the DNA damage and spindle checkpoint in response to replication stress induced by topoisomerase I inhibitor. Mol Cell Biol 25: 7889–7899. 16107732 10.1128/MCB.25.17.7889-7899.2005 PMC1190313

[14] Royou A, Macias H, Sullivan W (2005) The Drosophila Grp/Chk1 DNA damage checkpoint controls entry into anaphase. Curr Biol 15: 334–339. 15723794 10.1016/j.cub.2005.02.026

[15] Fong H, Hurley J, Hopkins R, Miake-Lye R, Johnson M, et al (1986) Repetitive segmental structure of the transducin b subunit: homology with the CDC4 gene identification of related mRNAs. Proc Natl Acad Sci USA 83: 2162–2166. 3083416 10.1073/pnas.83.7.2162 PMC323251

[16] Goodman O, Krupnick J, Gurevich V, Benovic J, Keen J (1997) Arrestin/clathrin interaction. Localization of the arrestin binding locus to the clathrin terminal domain. J Biol Chem 272: 15017–15022. 9169477 10.1074/jbc.272.23.15017

[17] terHaar E, Musacchio A, Harrison S, Kirchhausen T (1998) Atomic structure of clathrin: a β-propeller terminal domain joins an a zigzag linker. Cell 95 563–573. 9827808 10.1016/s0092-8674(00)81623-2 PMC4428171

[18] Neer E, Schmidt C, Nambudripad R, Smith T (1994) The ancient regulatory-protein family of WD-repeat proteins. Nature 371: 297–300. 8090199 10.1038/371297a0

[19] Loewith R, Jacinto E, Wullschleger S, Lorberg A, Crespo J, et al (2002) Two TOR complexes, only one of which is rapamycin sensitive, have distinct roles in cell growth control. Mol Cell 10: 457–468. 12408816 10.1016/s1097-2765(02)00636-6

[20] Roberg K, Bickel S, Rowley N, Kaiser C (1997) Control of amino acid permease sorting in the late secretory pathway of Saccharomyces cerevisiae by SEC13, LST4, LST7 and LST8. Genetics 147: 1569–84. 9409822 10.1093/genetics/147.4.1569 PMC1208332

[21] Kemp J, Balasubramanian M, Gould K (1997) A wat1 mutant of fission yeast is defective in cell morphology. Mol Gen Genet 254: 127–138. 9108274 10.1007/s004380050400

[22] Ochotorena I, Hirata D, Kominami K, Potashkin J, Sahin F, et al (2001) Conserved Wat1/Pop3 WD-repeat protein of fission yeast secures genome stability through microtubule integrity and may be involved in mRNA maturation. J Cell Sci 114: 2911–2920. 11686295 10.1242/jcs.114.16.2911

[23] Kominami K, Toda T (1997) Fission yeast WD-repeat protein Pop1 regulates genome ploidy through ubiquitin-proteasome-mediated degradation of the CDK inhibitor Rum1 and the S-phase initiator Cdc18. Genes Dev 11: 1548–1560. 9203581 10.1101/gad.11.12.1548

[24] Yang H, Rudge D, Koos J, Vaidialingam B, Yang H, et al (2013) mTOR kinase structure, mechanism and regulation. Nature 497: 217–224. 23636326 10.1038/nature12122 PMC4512754

[25] Moreno S, Klar A Nurse P (1991) Molecular genetic analysis of fission yeast Schizosaccharomyces pombe. Methods Enzymol 194: 793–823. 10.1016/0076-6879(91)94059-l 2005825

[26] Hagan IM, Hyams JS (1988) The use of cell division cycle mutants to investigate the control of microtubule distribution in the fission yeast Schizosaccharomyces pombe. J. Cell Sci. 89: 343–357. 10.1242/jcs.89.3.343 3198697

[27] Sazer S, Sherwood S (1990) Mitochondrial growth and DNA synthesis occur in the absence of nuclear DNA replication in fission yeast. J Cell Sci 97: 509–516. 2074269 10.1242/jcs.97.3.509

[28] Sali A, Blundell T (1993) Comparative protein modelling by satisfaction of spatial restraints. J Mol Biol 234: 779–81. 8254673 10.1006/jmbi.1993.1626

[29] Pettersen E, Goddard T, Huang C, Couch G, Greenblatt D, et al (2004) UCSF Chimera–a visualization system for exploratory research and analysis. J Comput Chem 25: 1605–1612. 15264254 10.1002/jcc.20084

[30] Gouet P, Courcelle E, Stuart D, Metoz F (1999) ESPript: analysis of multiple sequence alignments in PostScript. Bioinformatics 15: 305–8. 10320398 10.1093/bioinformatics/15.4.305

[31] Yadav S, Verma S, Ahmed S (2011) DNA topoisomerase II mutant activates DNA damage checkpoint protein kinase Chk1 in fission yeast S. pombe. Genet Res 93: 275–283. 10.1017/S0016672311000188 21767457

[32] Alfa C, Fantes P, Hyams J, McLeod M, Warbrick E (1993) Experiments with Fission Yeast. Cold Spring Harbor Laboratory, Cold Spring Harbor, NY.

[33] Zamore P, Patton J, Green M (1992) Cloning and domain structure of the mammalian splicing factor U2AF. Nature 355: 609–614. 1538748 10.1038/355609a0

[34] Potashkin J, Naik K, Wentz-Hunter K (1993) U2AF homolog required for splicing in vivo. Science 262: 573–575. 8211184 10.1126/science.8211184

[35] Takahashi K, Yamada H, Yanagida M (1994) Fission yeast minichromosome loss mutants mis cause lethal aneuploidy and replication abnormality. Mol Biol Cell 5: 1145–1158. 7865880 10.1091/mbc.5.10.1145 PMC301137

[36] Chen E, Kaiser C (2003) LST8 negatively regulates amino acid biosynthesis as a component of the TOR pathway. J Cell Biol 161: 333–347. 12719473 10.1083/jcb.200210141 PMC2172900

[37] Zhai Y, Borisy G (1994) Quantitative determination of the proportion of microtubule polymer present during the mitosis-interphase transition. J Cell Sci 107: 881–890. 8056844 10.1242/jcs.107.4.881

[38] Wise D, Cassimeris L, Rieder C, Wadsworth P, Salmon E (1991) Chromosome fiber dynamics and congression oscillations in metaphase PtK2 cells at 23°C. Cytoskel. 18: 131–142. 10.1002/cm.970180208 2013109

[39] Zachos G, Black E, Walker M, Scott M, Vagnarelli P, et al (2007) Chk1 is required for spindle checkpoint function. Dev Cell 12: 247–260. 17276342 10.1016/j.devcel.2007.01.003 PMC7115955

[40] Hayashi T, Hatanaka M, Nagao K, Nakaseko Y, Kanoh J, et al (2007) Rapamycin sensitivity of the Schizosaccharomyces pombe tor2 mutant and organization of two highly phosphorylated TOR complexes by specific and common subunits. Genes Cells 12: 1357–1370. 18076573 10.1111/j.1365-2443.2007.01141.x

[41] Heichman A, Roberts M (1996) The yeast *CDC16* and *CDC27* genes restrict DNA replication to once per cell cycle. Cell 85: 39–48. 8620535 10.1016/s0092-8674(00)81080-6

[42] Singer D, Manning M, Formosa T (1996) Coordinating DNA replication to produce one copy of the genome requires genes that act in ubiquitin metabolism. Mol Cell Biol 16: 1356–1366. 8657109 10.1128/mcb.16.4.1356 PMC231120

[43] Chan M, Botstein D (1993) Isolation and characterization of chromosome-gain and increase-in-ploidy mutants in yeast. Genetics 135: 677–691. 8293973 10.1093/genetics/135.3.677 PMC1205712

[44] Vallen A, Scherson Y, Roberts T, van Zee K, Rose D (1992) Asymmetric mitotic segregation of the yeast spindle pole body. Cell 69: 505–515. 1581964 10.1016/0092-8674(92)90451-h

[45] McGrew T, Goetsch L, Byers B, Baum P (1992) Requirement for *ESP1* in the nuclear division of Saccharomyces cerevisiae. Mol Biol Cell 3: 1443–1454. 1493337 10.1091/mbc.3.12.1443 PMC275712

[46] Pinto I, Winston F (2000) Histone H2A is required for normal centromere function in Saccharomyces cerevisiae. EMBO J 19: 1598–1612. 10747028 10.1093/emboj/19.7.1598 PMC310229

[47] Ren Q, Li R, Dicker A, Wang Y (2005) CHK1 Affects Cell Sensitivity to Microtubule -Targeted Drugs. J Cell Physiol 203: 273–276. 15389625 10.1002/jcp.20222

[48] Ahmed S, Palermo C, Wan S, Walworth N (2004) A novel protein with similarities to Rb Binding Protein 2 compensate for the loss of Chk1 function and affects histone modification in fission yeast. Mol. Cell. Biol. 24: 3660–366. 10.1128/MCB.24.9.3660-3669.2004 PMC387755 15082762

[49] Ahmed S, Dul B, Qiu X, Walworth N (2007) Msc1 Acts through Histone H2A.Z to Promote chromosome stability in Schizosacchromyces pombe. Genetics 177: 1487–1497. 17947424 10.1534/genetics.107.078691 PMC2147988

